# Deep neural network training method based on vectorgraphs for designing of metamaterial broadband polarization converters

**DOI:** 10.1038/s41598-023-32142-1

**Published:** 2023-03-27

**Authors:** Jiale Gao, Chunjie Feng, Xingyi Wu, Yanghui Wu, Xiaobo Zhu, Daying Sun, Yutao Yue, Wenhua Gu

**Affiliations:** 1 School of Microelectronics (School of Integrated Circuits), Nanjing University of Science and Technology, Nanjing, 210094 China; 2 Institute of Deep Perception Technology, Wuxi, 214000 China

**Keywords:** Optical materials and structures, Computer science

## Abstract

In this work, we proposed a method of extracting feature parameters for deep neural network prediction based on the vectorgraph storage format, which can be applied to the design of electromagnetic metamaterials with sandwich structures. Compared to current methods of manually extracting feature parameters, this method can automatically and precisely extract the feature parameters of arbitrary two-dimensional surface patterns of the sandwich structure. The position and size of surface patterns can be freely defined, and the surface patterns can be easily scaled, rotated, translated, or transformed in other ways. Compared to the pixel graph feature extraction method, this method can adapt to very complex surface pattern design in a more efficient way. And the response band can be easily shifted by scaling the designed surface pattern. To illustrate and verify the method, a 7-layer deep neural network was built to design a metamaterial broadband polarization converter. Prototype samples were fabricated and tested to verify the accuracy of the prediction results. In general, the method is potentially applicable to the design of different kinds of sandwich-structure metamaterials, with different functions and in different frequency bands.

## Introduction

Electromagnetic (EM) metamaterials can effectively manipulate the propagation, polarization, and wavefront of EM waves^1,2^, and have been widely used in many applications, including absorbers, EM shielding, and polarization converters^3,4^. The EM metamaterial usually adopts a typical sandwich structure, including a 2D conductive surface pattern, an intermediate dielectric layer, and an underlying conductive ground layer. The main design parameters lie in the various geometrical parameters of the 2D conductive surface pattern, plus two parameters of the dielectric layer, i.e., the dielectric constant and the thickness. The traditional EM metamaterial design process usually includes empirical model design, parameter scanning and optimization with the help of commercial software simulation, which usually requires a lot of computing resources and time.

Artificial neural networks have shown many advantages in areas including prediction and clustering^5^. Recently, many groups are also exploring the use of artificial neural networks for the inverse design of EM metamaterials^6–10^. J. Wang, et al. proposed a data cropping algorithm to design a low-profile, broad transmission-band absorption frequency selective transmission (AFST) subsurface pattern^11^. Zhu et al. used CNN networks to extract electromagnetically induced transparency (EIT) metasurface feature parameters^12^. Chang et al. successfully predicted metal mesh shielding effectiveness curves using BP networks and further used them to guide the design of surface patterns for given shielding effectiveness requirements^13^. In all these works, feature parameters of the metamaterial structures were manually extracted to guide the neural network training.

A general solution of feature extraction is to directly employ pixel graphs to represent the surface pattern, but it requires a large number of computing resources. Hodge et al. used deep convolutional generative adversarial networks (DC-GANS) to guide the design of polarization converters, using metamaterial cells in over 150 reflectance array configurations as the training dataset for predicting the relationship between surface pattern parameters and the reflectance spectra of the two polarizations^14^. Liu et al. used “1” and “0”to indicate whether metal is attached to a 16*16 square sub-block of the surface pattern, and 70,000 coding patterns were used to train the neural network to obtain the reflection phase at 10 GHz^15^. This feature extraction approach based on pixel graphs is very successful, but requires a huge amount of data and computational resources, and the optimization can be pretty complex. This work suggests using the vectorgraph to extract feature parameters from surface patterns of the sandwich-structured metamaterial, which can be a general and efficient method for neural network design of metamaterial.

**Figure 1:**
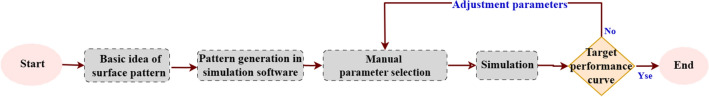
Flowchart of the conventional manual optimization method.

**Figure 2:**
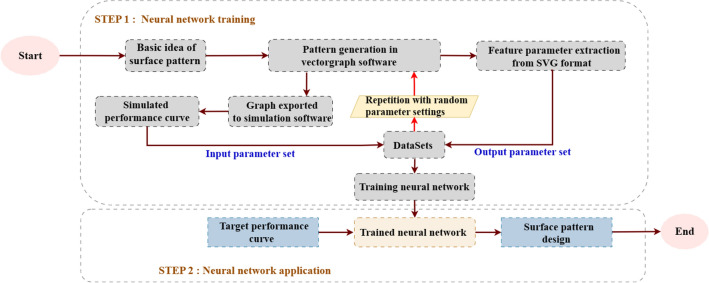
Flowchart of vectorgraph parameter extraction method design process.

For comparison, we show the flowchart of the conventional metamaterials design process (Fig. 1), as well as that of the proposed method (Fig. 2). It can be seen that to propose the basic idea of surface pattern is the first step for both methods. The idea can come from theoretical directions, or literature survey results, and so on. The design process itself does not make any limitation to the surface pattern design, but it is necessary to come up with some basic pattern design first to avoid very complex optimization process. With the basic design of surface pattern, the vector graph can be directly generated using vectorgraph software (CAD, Solidworks, etc.), as shown in Fig. 2.

Meanwhile. To illustrate the contribution of this manuscript, table 1 makes a simple comparison of the proposed method to the conventional metamaterial design method as well as two other AI design methods.

**Table 1:** Comparison of the proposed method to the conventional metamaterial design method as well as two other AI design methods.

Method	Neural network input v.s. output	Suitable for complex surface pattern design	Computer resource needed	Progress can be automated	Can cover all kinds of pattern varieties
Conventional manual optimization method	–	No	Small	No	No
Pixel graph parameter extraction method	Target performance curves v.s. Pixel graph of the surface pattern	Yes	Huge	No	Yes
Manual parameter extraction method	Target performance curves v.s. Feature parameters manually extracted from the surface pattern	No	Medium	No	Not guaranteed
Our work: vectorgraph parameter extraction method	Target performance curves v.s. Feature parameters automatically extracted from the vector graph of the surface pattern	Yes	Medium	Yes	Yes

## Vectorgraph feature-extraction method

**Figure 3:**
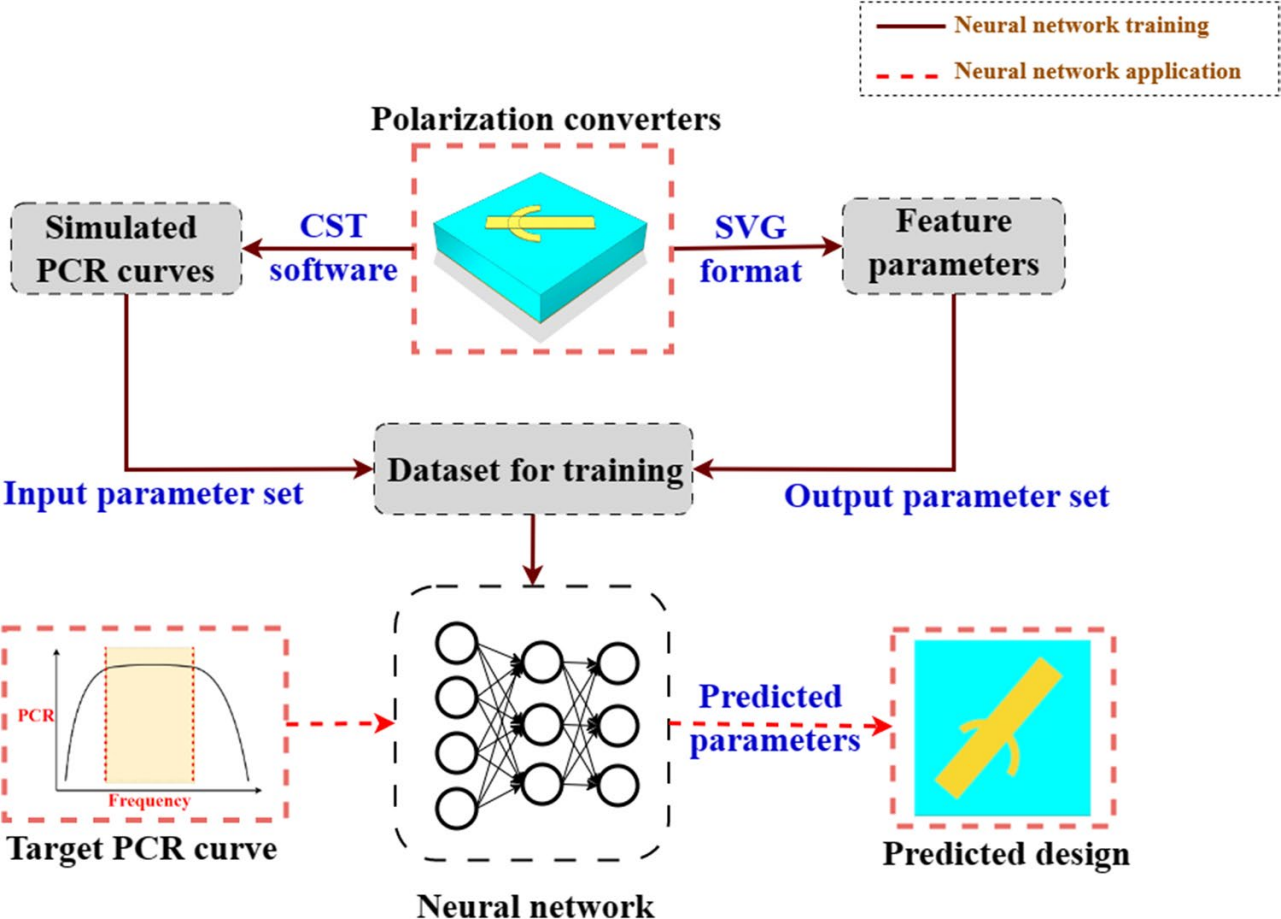
Block diagram illustrating the system of the proposed deep neural network training method based on vectorgraphs for designing metamaterial broadband polarization converters.

The vectorgraph is a well-established digital storage technology for 2D graphs. It features high information density and easy transformation, and automatically extracts key feature parameters of a given graph, including the location, shape, size, contour, color, and other features. The fact that vector graphics are not distorted with the change of scale is another important feature that is superior to pixel graphics. There are several mature vectorgraph storage formats for selection. For example, the Scalable Vector Graphics (SVG) format can enable dynamic description and employ descriptive language in text format to describe the visual content. From the SVG format of a given surface pattern, the feature parameters can be directly extracted to fully describe the 2D pattern without any distortion. Part of unnecessary information such as color can be directly removed or replaced by other parameters such as surface resistance if needed. Therefore, the manual and random selection of feature parameters can be replaced by an automatic and determined method, where the feature parameters can be automatically extracted from the SVG file by a simple computer code under certain rules, as will be illustrated in this report.

Assume N feature parameters are extracted from the SVG file, all we need to do is to add two parameters of the dielectric layer: the thickness t and the permittivity ${{\varepsilon }_{\text {r}}}$, and the metamaterial design can be fully described by these [N+12] parameters. We can further correlate the desired EM properties of the metamaterial to the [N+12] parameters for data training. After sufficient and correct data training, the deep neural network can predict the EM properties of any given metamaterial, or backwardly design the metamaterial structure according to the required EM properties. Figure 3 shows the block diagram of the deep neural network training method based on vectorgraphs for designing metamaterial broadband polarization converters.

**Figure 4:**
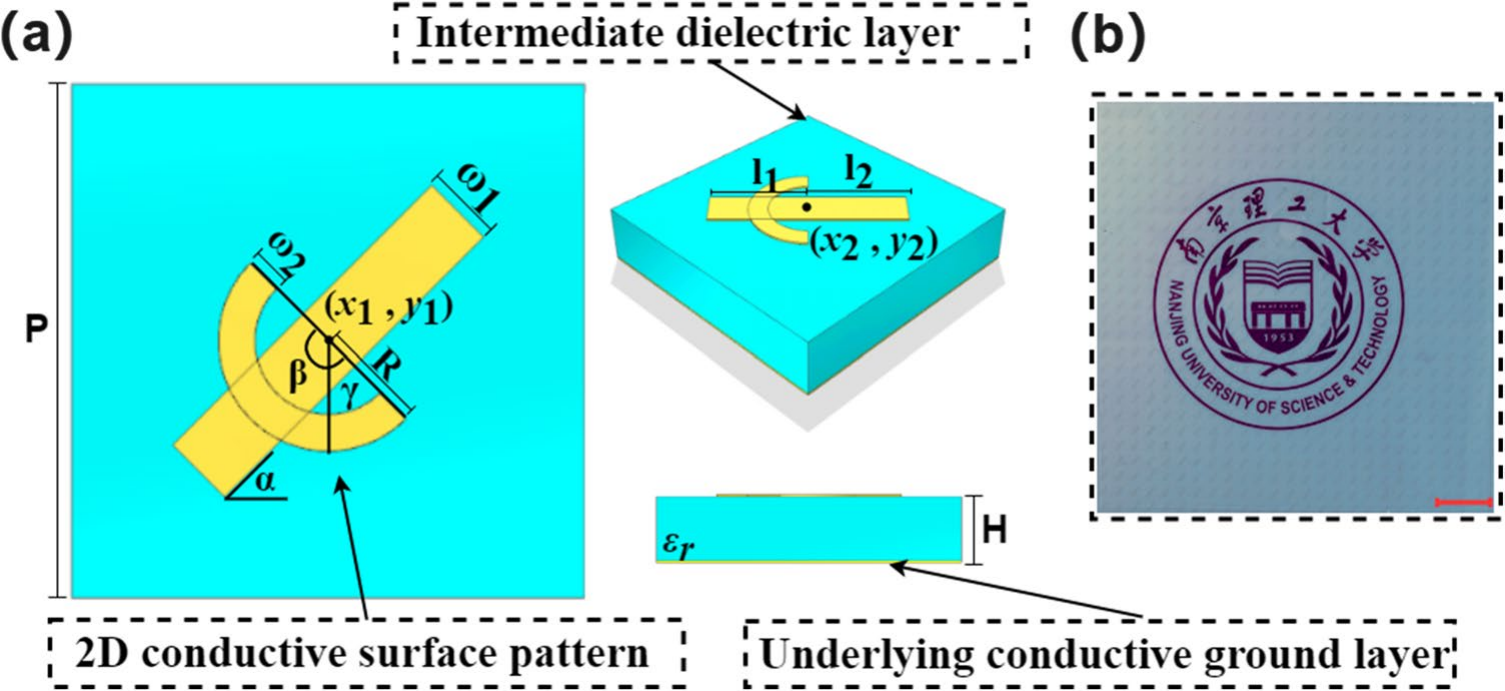
(**a**) Schematic diagram and parameter definition of the surface pattern and the metamaterial. (**b**) Photo of Example 1, on top of a piece of white paper with the university logo printed on it to show the transparency of the sample (scale bar: 2cm).

Of course, there are almost infinite surface pattern design possibilities, so there should be certain limits of the surface pattern for practical and meaningful data training. This work demonstrates a simple surface pattern as the example for the design of a transparent reflection-type broadband polarization converter, which is a combination of a line and an arc, as illustrated in Fig. 3. The classical slash structure is used to implement the polarization conversion function as an example to specify how the parameters can be extracted and used for neural network prediction by means of a vectorgraph. In order to increase the complexity of the structure, a circular structure is added to the basic slash structure. The SVG format description of the vectorgraph is very concise. Using the structure shown in Fig. 4 as an example, the feature parameters can be directly extracted from the SVG file, as listed in Table 2.

**Table 2:** SVG format interpretation and parameter extraction.

Source file	Description	Feature parameter extraction
<svg width=’100%’ height=’100%’ version= "1.1" xmlns=’http://wwww.w3.org/2000/svg’> $<\text {title}>$Sandwich Structure$</\text {title}>$	File head	–
$<\text {rect}$ x= "0" y= "0" width="7" height = "7" fill="RGB (0,255,255) "$> </\text {rect}>$	Rectangular period structure positioned at (0,0), width 7mm, height 7mm, color defined as RGB (0,255,255), representing cyan	${{x}_{0}}=0,{{y}_{0}}=0$ $width=0$ $height=0$ $RGB\left( 0,255,255 \right)$
$<\text {rect}$ x= "2" y= "1" width="1" height = "5" transform= "translate (3.5, 3.5) rotate (45)" fill="RGB (255,255,0) "> </rect>	Left-up corner of the rectangle positioned at (2,1), 1mm wide, 5mm high, rotated 45$^\circ$ around the (3.5,3.5) point, the color defined as RGB (255,255,0), representing yellow	${{x}_{3}}=2,{{y}_{3}}=1$ ${{\omega }_{1}}=1,L=5$ ${{x}_{1}}=3.5,{{y}_{1}}=3.5$ $\alpha =45{}^\circ$ $RGB\left( 255,255,0 \right)$
$<\text {path}$ d="M 3.5 1.5 A 3.5 3.5, 135, 0, 1, 5.5, 3.5 stroke-width="0.5" fill="RGB (255,255,0) ">	The arc starts from (3.5, 1.5), centered at (3.5, 3.5), rotated 135$^\circ$ clockwise, and ends at (5.5, 3.5); the arc width is 0.5mm, the color defined as RGB (255, 255, 0) representing yellow	${{x}_{4}}=3.5,{{y}_{4}}=1.5$ ${{x}_{2}}=3.5,{{y}_{2}}=3.5$ $\gamma =135,c=1$ $t=0$ ${{x}_{5}}=5.5,{{y}_{3}}=3.5$ ${{\omega }_{2}}=0.5$ $RGB\left( 255,255,0 \right)$
$</\text {svg}>$	File end	–

In principle, the feature parameters extracted in Table 1 can be directly used for the DNN training. However, for simplicity and better adaptability to the simulation software parameter setting, the feature parameters are further processed. First, all color information is not needed. Second, a fixed rectangle period is set for the data training, so all the parameters related to the periodic structure can be ignored. Third, since both $\left( {{x}_{1}},{{y}_{1}} \right)$ and $\left( {{x}_{3}},{{y}_{3}} \right)$ can be used to describe the location of the rectangle, one representing the center and the other representing the left-up corner, only one is needed, and $\left( {{x}_{1}},{{y}_{1}} \right)$ is kept. Fourth, it is more convenient to use the arc radius R and arc angle $\gamma$ to describe the arc in the simulation software, so the following translation equations are used:

1 $$\begin{aligned} \begin{aligned} \left\{ \begin{matrix} {{x}_{4}}={{x}_{2}} \\ {{y}_{4}}={{y}_{2}}-R \\ {{x}_{5}}={{x}_{2}}+R\sin \gamma \\ {{y}_{5}}={{y}_{2}}-R\cos \gamma \\ \end{matrix} \right. \end{aligned} \end{aligned}$$

Therefore, altogether feature parameters can be extracted based on the SVG file, and design parameters are used for the metamaterial description, as shown in Table 3. The value range of each parameter is also listed in Table 3.

**Table 3:** Extracted feature parameters and their value ranges.

Parameters	Value Range	Parameters	Value range
H(mm)	1–3	${{l}_{2}}$ (mm)	2–5
${{\varepsilon }_{\text {r}}}$	3–6	${{x}_{2}}$	2–5
${{x}_{1}}$	1–6	${{y}_{2}}$	2–5
${{y}_{1}}$	1–6	${{w}_{2}}$(mm)	0.5–1.5
${{w}_{1}}$(mm)	0.3–1	$\beta$ ($^\circ$)	60–360
$\alpha$($^\circ$)	15–75	$\gamma$ ($^\circ$)	15–75
${{l}_{1}}$(mm)	2–5	R(mm)	1–6

The polarization conversion rate (PCR) spectra in (10–20) GHz of the metamaterial structure were acquired via CST software simulation (Version: CST Studio Suite 2020, https://www.3ds.com/) as the output set, and 450 sets of feature parameters were randomly chosen within the given range as the training data set, among which 15 sets were taken as the validation set.

## Deep neural network optimization

**Figure 5:**
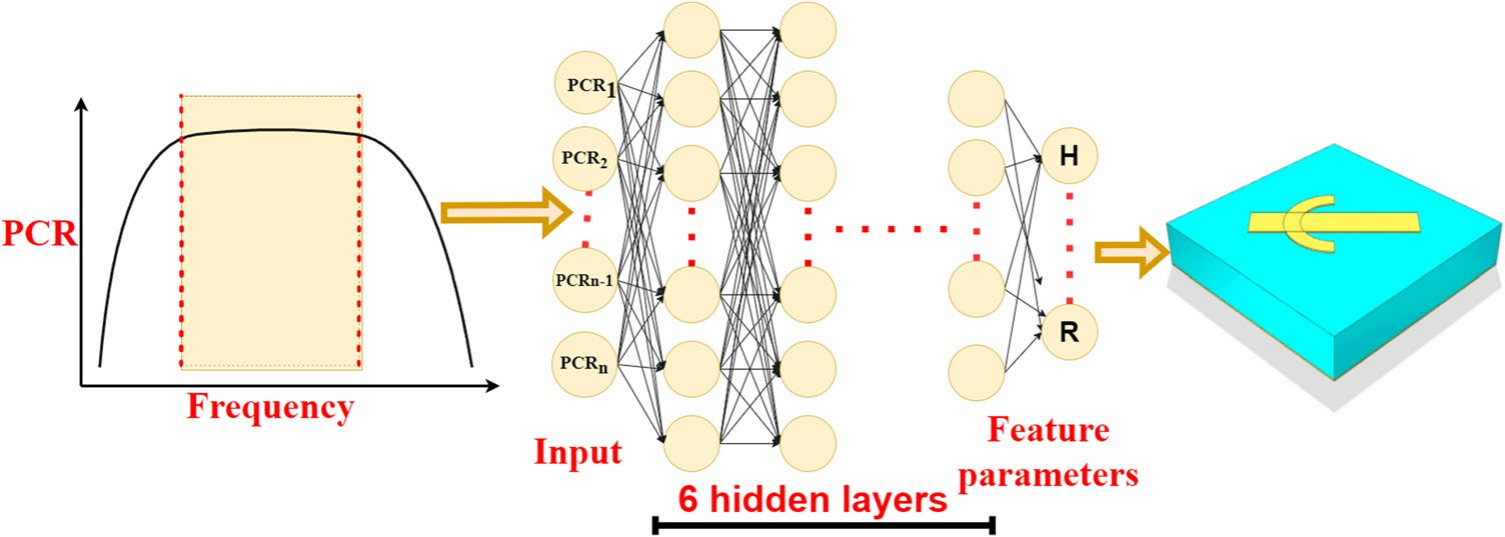
Illustration of the deep neural network (DNN) model. The target PCR spectrum is used as the input layer at the left end, the 7-layer DNN network is in the middle, and the metamaterial structure parameters is at the right side as the output.

A 7-layer deep neural network (DNN) was used for the metamaterial training and prediction. Figure 5 depicts the network topology of the DNN employed in this work. The neurons in layer *i* are connected to the neurons in layer $i+1$, and the successive layers are completely interconnected. The primary activation functions used in this DNN are ELU and Tanh.

The loss function used in this work is the mean square error (MSE) loss function, which is given by:

2 $$\begin{aligned} L\left( Y|f\left( x \right) \right) =\frac{1}{n}\sum \limits _{i=1}^{n}{{{\left( {{Y}_{i}}-f({{x}_{i}}) \right) }^{2}}} \end{aligned}$$

where ${{Y}_{i}}$ is the sample data and $f({{x}_{i}})$ is the fitted data. The MSE loss function is used to measure the difference between the predicted value and the actual value, and the squared loss should be minimized. The smaller the value of MSE, the higher the accuracy of the prediction model. In addition, in order to prevent overfitting, and to avoid the lack of model versatility due to the small amount of data, the L2 regularization is used to limit the weights, which is given by:

3 $$\begin{aligned} {{J}_{loss}}=MSE+\frac{1}{m}\frac{\lambda }{2}\sum \limits _{i=1}^{n}{{{W}_{i}}^{2}} \end{aligned}$$

where $\lambda$ is the weight of the L2 regularization and is ${{W}_{i}}$ the weight vector of the network. The degree of regularization is measured by the L2 regularization factor. Before each activation function layer, a BN (Batch Normalization) layer is added to speed up network training and increase the model stability. Adam (Adaptive Moment Estimation) method is selected as the optimizer. The learning rate decay and early stop methods are also employed in order to acquire the best model. All the above skills together form the primary architecture of the DNN used in this work.

**Figure 6:**
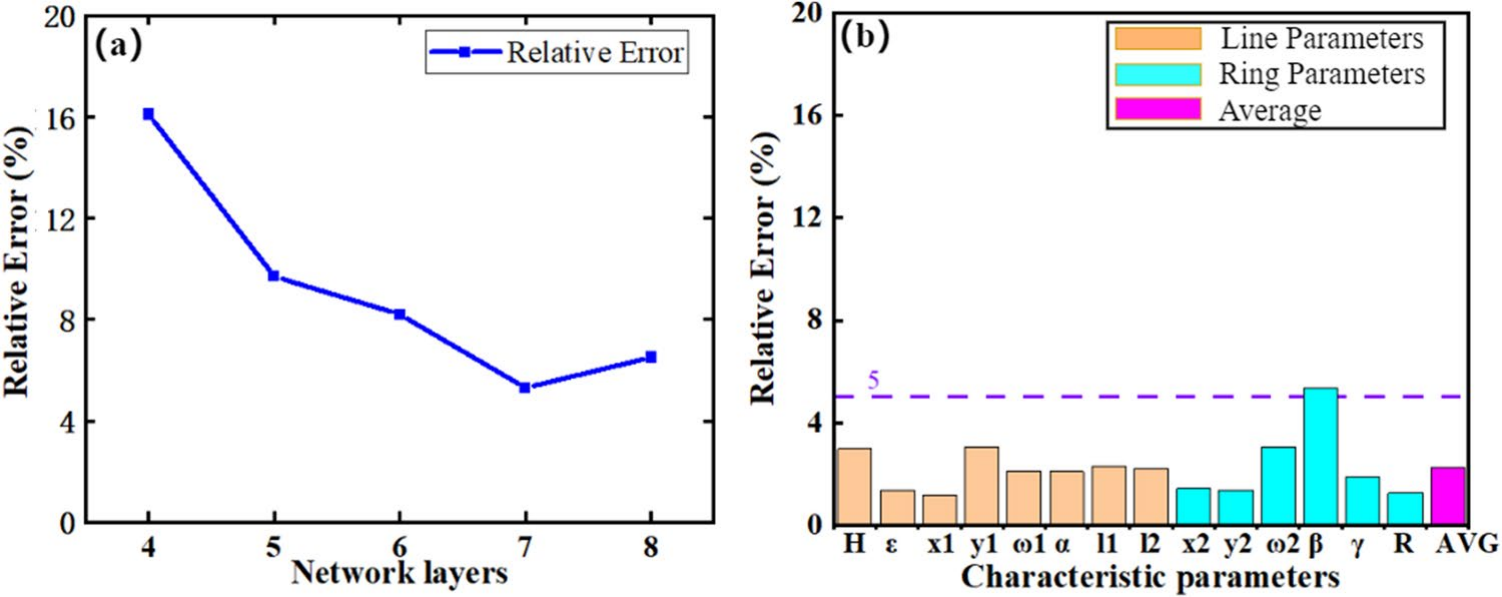
(**a**) The effect of the number of network layers on the network prediction error(based on ReLU); (**b**) Average prediction error of the 14 feature parameters given by the validation set (15 data sets). (Plotted in OriginPro 2021).

The Intel(R) Core (TM) i9-10980XE CPU architecture served as the neural network operating environment. The input is a 334-point data set sampled with 0.03 GHz step in (10–20) GHz. As shown in Fig. 6, the average prediction error of the feature parameters was 2.3%, as given by the validation set (15 data sets), under the following conditions: the number of network neurons was (512, 512, 512, 512, 256, 64), the learning rate $\alpha$ was 0.04, Adam’s default parameters were utilized, the L2 regularization coefficient was 0.05, and the early stopping factor was 1200. The sample size was set to be 205 mm*205 mm, the unit surface pattern period was 7 mm, the surface resistance was (6–8) $\Omega /sq$, and the thickness of the bottom conductive ground layer was 0.125 nm.

**Table 4:** Comparison of average errors with different network layers and activation functions.

Network layers	Average of error (%)	Activation function	Average of error (%)
8	6.50	ReLU	5.30
7	5.30	ELU	3.20
6	8.20	Tanh	8.90
5	9.70	ReLU+ELU	4.20
4	16.10	ELU+Tanh	2.30

In this study, the optimization process of the deep neural network go through the following steps:

Step 1, the number of levels of the network needs to be determined, and the typical values of the other parameters of the deep neural network are selected and kept constant when evaluating the impact of the number of layers on the network training. When optimizing the number of layers of the network, the activation functions were all ReLU functions, and the number of neurons in each layer was 512. This number was chosen because the input dimension was 334, so the nearest power of 2 larger than 334 was selected. At the same time, the L2 parameter was chosen to be 0.2. For optimization, the number of network layers started from 4 and the optimization step was 1 until the inflection point of the error function appeared. It can be seen from Fig. 6a that the average error of the network output decreases continuously as the number of network layers increases, until it reaches the lowest value where the number of network layers is 7; then the error bounces back with further increased number of network layers, which is probably due to network overfitting.

Step 2, in the above process, there are three main options of activation functions in this paper, which are ReLU function, ELU function and Tanh function. The error functions of the three activation functions under 7 network layers are tested separately, and the results are listed in Table 4. It can be found that the ELU function has the smallest error of 3.2%.

**Table 5:** Design of three samples with typical target PCR spectra.

Parameter	Example 1	Example 2	Example 3	Example 4	Example 5
Target parameters					
Bandwidth type	Broadband	Broadband	Broadband	Single point	Single point
PCR	0.96	0	0.96	1	1
${{\varepsilon }_{\text {r}}}$	3 (PET)	5.5 (glass)	5.5 (glass)	4.8	3.6
Center frequency	15 GHz	15 GHz	15 GHz	15 GHz	18 GHz
Predicted parameters					
Predicted pattern					
H (mm)	2.3	2.5	2.02	2.4	2.7 [t]
${{x}_{1}}$	4.36	5.52	5.37	2.31	4.03
${{y}_{1}}$	4.65	5.51	4.59	2.33	1.82
${{w}_{1}}$(mm)	1.18	1	1.12	1.2	0.3
$\alpha$($^\circ$)	40.57	45	51.68	44.2	30.8
${{l}_{1}}$(mm)	2.94	1.8	2.27	3.8	2.2
${{l}_{2}}$(mm)	3.54	0.5	3.01	0.5	0.2
${{x}_{2}}$	3.89	4.48	4.27	3.92	3.53
${{y}_{2}}$	3.73	4.53	4.07	3.88	2.56
${{w}_{2}}$(mm)	0.64	1	1.11	1.1	1.2
$\beta$($^\circ$)	159.99	149.3	163.48	143.5	277.6
$\gamma$($^\circ$)	37.795	29.65	39.9	44.8	35.8
R(mm)	2.98	2	2.8	4.2	2.63 [b]

Step 3, other hyperparameters can be further optimized, including the number of neurons at the end of the output layers and the value of L2 regulation parameter, and the lowest error of 2.86% was obtained with final number of neurons of (512, 512, 512, 512, 256, 64) and L2 parameter as 0.04.

Step 4, an additional attempt was made to see if the error could be reduced by performing a combination of activation functions based on the activation function ELU, which is the activation function with the lowest error in the first step. We tried "ELU+ReLU", "ReLU+ELU", "Tanh+ELU" and "ELU+Tanh ", as well as the adjustment of different activation functions with different number of network layers, the error of the network varies considerably.

Finally, after the optimization process described above, this study achieved an average error of 2.3% (average for the 14 output parameters of the network), by building up a 7-layer DNN network with the number of neurons (512, 512, 512, 512, 256, 64, 14), where the first four layers of the network activation function are ELU functions, the last three layers are Tanh functions, and the L2 regularization parameter is 0.04.

**Figure 7:**
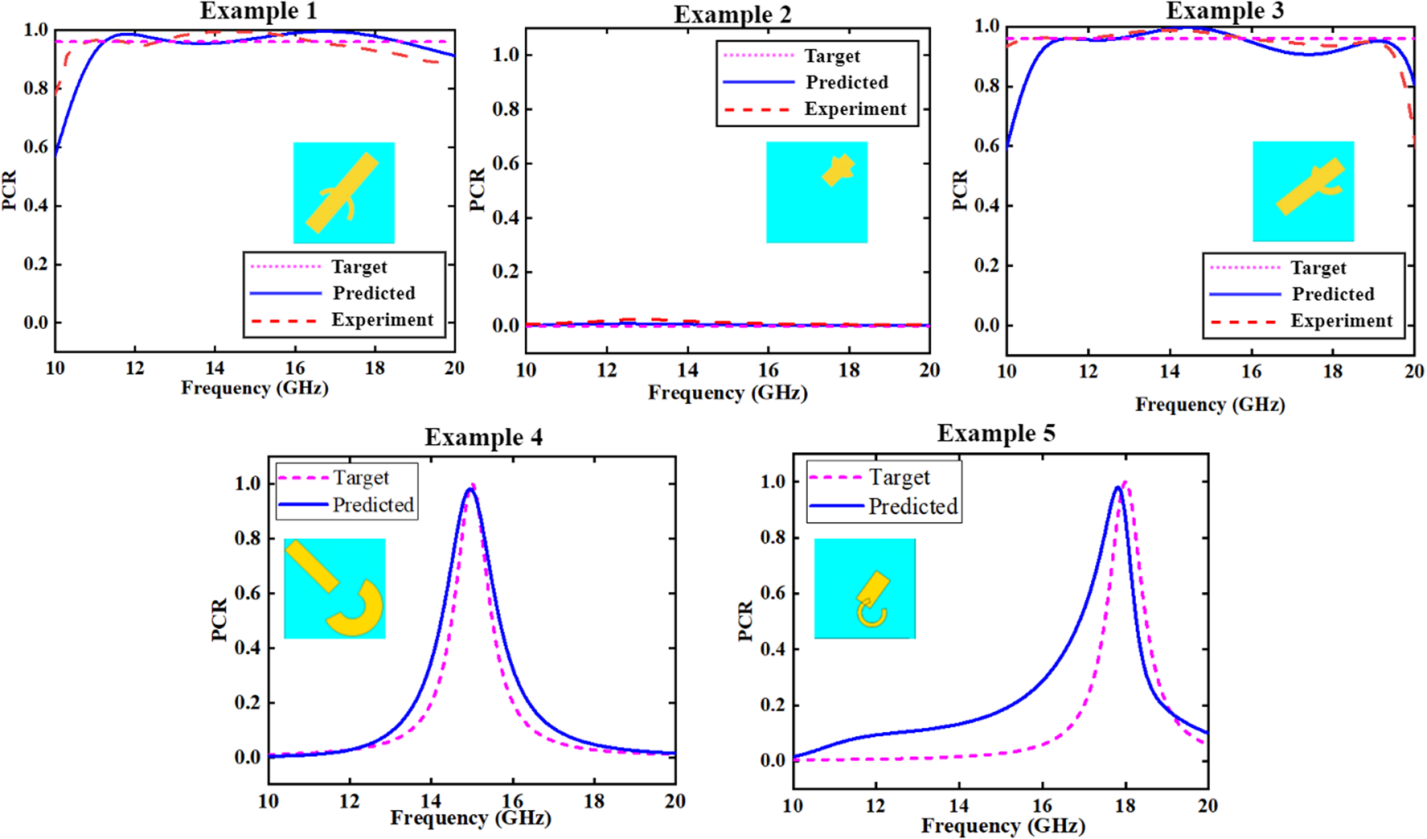
Comparison between target data, predicted data, and experiment data for broadband frequency of Examples 1, 2, and 3; Comparison between target data and predicted data for single frequency points of Example 4 and Example 5. (Plotted in OriginPro 2021).

Three typical target PCR spectra were chosen to verify the metamaterial design method, and three samples were made correspondingly, as shown in Table 5 (Example 1, 2, and 3). Here the dielectric constant 3 corresponds to the dielectric material polyethylene terephthalate (PET), and the dielectric constant 5.5 corresponds to the dielectric material glass, both are optically transparent. The transparent conductive material for the surface pattern and ground layers was chosen to be indium tin oxide (ITO), and the surface layer was patterned using yellow-light etching technology. The samples were fabricated and measured, as introduced in detail in the next section. The target, predicted, and measured PCR curves of the three samples are plotted together for comparison, as shown in Fig. 7. Calculation shows that, in the (10–20) GHz range, the average error of the experiment value versus the prediction value was 0.14%, 0.001%, and 0.1%, respectively, for the three samples.

What is more, the proposed method can also be used to design single-frequency-point polarization converters at any given center frequency, as illustrated in Table 5 (Examples 4 and 5). Two center frequency points (15 GHz and 18 GHz) were randomly chosen, with the dielectric constants randomly set to be 4.8 and 3.6, respectively. The target single-frequency-point PCR spectra were artificially defined using the Origin function generator (Version: OriginPro 2021, https://www.originlab.com/), as shown in Fig. 7. The proposed method can predict the polarization converter design parameters precisely, as listed in Table 5 (Examples 4 and 5). The corresponding PCR curves at these given parameters can be simulated by CST (shown as Predicted curve), for comparison to the target curves (shown as Target curve), as shown in Fig. 7. The spectra matching is reasonably good. Due to the randomly chosen dielectric constants, it is difficult to find proper materials for sample fabrication.

## Experiment

**Figure 8:**
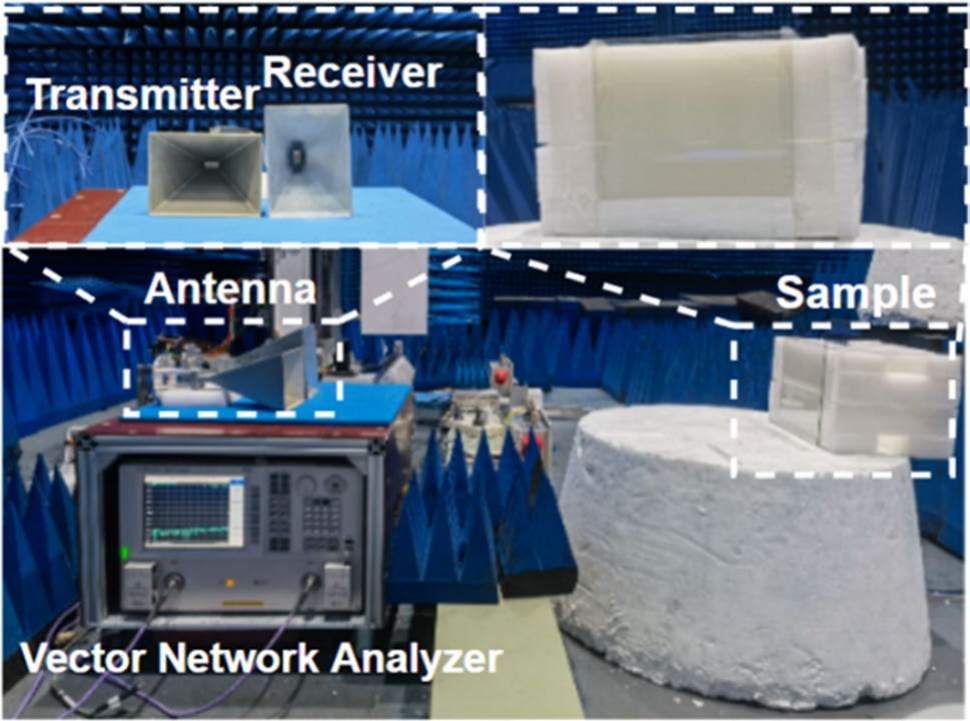
Measurement of the PCR spectra in the microwave darkroom.

The polarization conversion spectra of the samples were measured in a microwave darkroom with an N5244A PNA-X Network Analyzer (Agilent Technologies), as shown in Fig. 8. Two standard horn antennas were used as transmitter and receiver, respectively. The distance between the horn antenna and the sample satisfies the far-field condition. An aluminum foil with the same size as the samples were placed and measured at the same position as the reference reflection plane^16^.

**Figure 9:**
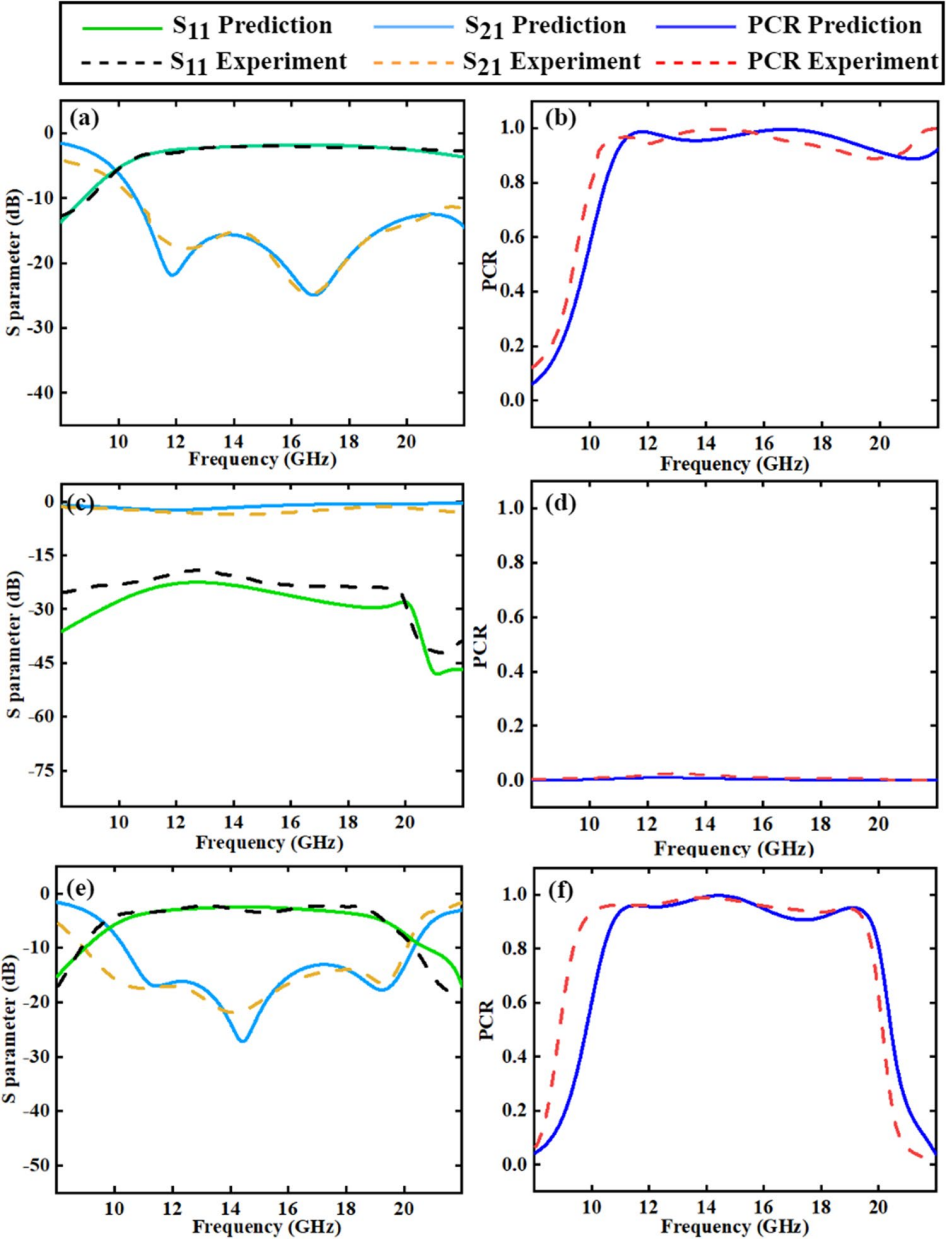
Comparison between the simulation results of the design target and experiment data, including S11, S21, and PCR spectrum which are represented by dotted Lines and lines. (**a**) (**c**) (**e**) are the S-parameter spectra of samples 1, 2, and 3, (**b**) (**d**) (**f**) are the PCR spectra of samples 1, 2, and 3, respectively. (Plotted in OriginPro 2021).

Figure 9 compares the simulated and measured S-parameter and PCR spectra of the samples, which match reasonably well. A more precise comparison can be run by calculating the average error and decision variance of the experiment value versus the prediction value. The decision variance can be calculated as:

4 $$\begin{aligned} {{R}^{2}}=1-FVU=1-\frac{\sum \nolimits _{n}{{{\left( {{y}_{i}}-{{y}_{p}} \right) }^{2}}}}{\sum \nolimits _{n}{{{\left( {{y}_{i}}-{\bar{y}} \right) }^{2}}}} \end{aligned}$$

where ${{y}_{i}}$ denotes the actual value, ${\bar{y}}$ is the average of the actual values, ${{y}_{p}}$ is the predicted value, and ${{R}^{2}}$ is referred to as the fraction of variance unexplained. The closer is to 1, the better the regression analysis is. Calculation shows that, in the (10–20) GHz range, the relative average error of the experiment value versus the prediction value is 0.14%, 0.001%, and 0.1%, respectively, for the three samples, and the decision variance is 0.92, 0.96, and 0.97, respectively. These statistical results indicate that the prediction is very successful. Possible reasons for the deviation might include: (1) the actual dielectric constant of the dielectric layer for PET/glass may deviate quite a bit from the set value of 3/5.5; (2) sample fabrication error; (3) measurement and data processing errors.

## Dissussion

In this work, a general design method is proposed and demonstrated to extract the feature parameters of a sandwich-structured EM metamaterial from the vectorgraph for DNN training and prediction. By employing a 7-layer DNN, this method was used to successfully design polarization converters with target PCR spectra, with an inaccuracy of 2.3% on the validation set. Three optically transparent broadband polarization converters were fabricated and measured for demonstration and verification. For the three samples, the average error of the experiment versus predicted values was only 0.14%, 0.0016%, and 0.1%, respectively; and the decision variance was 0.92, 0.96, and 0.97, respectively.

A comparison of this work to other reports on the neural-network-based design of metamaterial EM devices is shown in Table 6.

In summary, the proposed deep-neural network-training method based on vectorgraphs can express arbitrary complex two-dimensional patterns with a large number of effective feature quantities. What is more, thanks to the merits of vectorgrpahs, the designed patterns can be easily deformed, including scaling, rotating, shifting, and other deformations. It is a universal feature extraction method and is effectively applicable to the design of all kinds of metamaterial sandwich structures. The method allows design of complex surface patterns, and can be applied to the design of sandwich-structure metamaterial EM devices with different frequency bands and functions, providing a new technology path for the structural design of metamaterials.

**Table 6:** Comparison of current work on metamaterial device designed by neural network.

Type	Number of parameters	Neural network	Band	Realized/target$^{\textrm{a}}$	Relative error$^{\textrm{b}}$	Deformable$^{\textrm{c}}$	Method$^{\textrm{d}}$ / parameter extraction
Antenna^7^	4	DNN	5.8GHz	T = 92.6%/100%	N.A.	No	2/Manual
DLMP^8^	3	GRNN	(0.1–3)$\mu m$	Ab = 95 %/100%	N.A.	No	2/Manual
UWPA^10^	26	ANN	(8–12)$\mu m$	Ab = 94.1%/100%	7.6%	No	2/Manual
AFST^11^	10	DNN	(7.5–14) GHz	Ab = 75%/100%	N.A.	No	2/Manual
EIT^12^	5	CNN	(1.5–2.5) THz	Am = 0.98/1	3.4%	No	2/Manual
EM shielding^13^	3	DNN	(0–20) GHz	SE = − 20dB	17%	No	2/Manual
Phase modulation^15^	256	Resnet-101	10GHz	360$^{\circ }$Phase /360$^{\circ }$Phase	9.95%	No	1/N.A.
Polarization converters (this work)	14	DNN	(10–20)GHz (15,18) GHz	PCR =96%/100%	3.7%	Yes	3/Automatic

$^{\textrm{a}}$T: transmittance. Ab: Absorption.Am: Amplitude.SE: Shielding effectiveness.

$^{\textrm{b}}$Relative error between predicted target and simulation results. Calculating Equation: *Relative* $Error=\sqrt{MSE}/Range$.

$^{\textrm{c}}$Deformable: the pattern is scalable, rotatable, and shiftable.

$^{\textrm{d}}$1: Pixel graph parameter extraction method. 2: Manual parameter extraction method. 3: Vectorgraph parameter extraction method.

## Data Availability

The data sets that support the findings in this study are available from the corresponding author upon reasonable request.
